# From Research to Education: When Natural Teeth Are the Only Reference—Student Perceptions of PolyJet™ 3D-Printed Teeth in Endodontic Training

**DOI:** 10.3390/dj14060346

**Published:** 2026-06-05

**Authors:** Cláudia Barbosa, Tiago Reis, José B. Reis, Margarida Franco, Catarina Batista, Rui B. Ruben, Benjamín Martín-Biedma, José Martín-Cruces

**Affiliations:** 1 Endodontics and Restorative Dentistry Unit, School of Medicine and Dentistry, University of Santiago de Compostela, 15701 Santiago de Compostela, Spain; cbarbosa@ufp.edu.pt (C.B.); pepe3214@gmail.com (J.M.-C.); 2 FP-I3ID, FP-BHS, Faculty of Health Sciences, University Fernando Pessoa, 4200-150 Porto, Portugal; 3 RISE-Health, Faculty of Health Sciences, University Fernando Pessoa, Fernando Pessoa Teaching and Culture Foundation, 4200-150 Porto, Portugal; 4 CDRSP, Polytechnic University of Leiria, 2430-028 Marinha Grande, Portugal; margarida.franco@ipleiria.pt (M.F.); catarina.batista@ipleiria.pt (C.B.); rui.ruben@ipleiria.pt (R.B.R.); 5 Faculty of Engineering, University of Porto, 4200-465 Porto, Portugal; jose.b.reis05@gmail.com; 6 Oral Sciences Research Group, Endodontics and Restorative Dentistry Unit, School of Medicine and Dentistry, University of Santiago de Compostela, Health Research Institute of Santiago de Compostela (IDIS), 15706 Santiago de Compostela, Spain; benjamin.martin@usc.es

**Keywords:** endodontic training, PolyJet™, 3D printing, 3D-printed teeth, endodontics

## Abstract

**Objectives:** Commercial artificial teeth (AT) and three-dimensional printed teeth (3DPT) have been increasingly used in preclinical endodontic education; however, limitations regarding anatomical realism, tactile sensation, and procedural simulation continued to be reported. This study assessed students’ and evaluators’ perceptions regarding AT and PolyJet™ 3DPT fabricated with RGD525™, compared with natural teeth (NT), together with the quality of endodontic procedures performed using both artificial models. **Methods:** Undergraduate dental students with no previous experience using AT or 3DPT performed standardized endodontic procedures on both artificial models. Students and evaluators completed questionnaires regarding anatomical realism, tactile sensation, radiographic characteristics, educational applicability, and model preference. Procedural quality and errors were independently assessed radiographically by evaluators. **Results:** AT received more favorable perceptions regarding external anatomy, whereas 3DPT were more positively evaluated for internal anatomy, radiopacity, resistance of root canal walls and tactile sensation during instrumentation (*p* ≤ 0.002). NT remained the preferred training model, followed by 3DPT, while AT received the lowest preference ratings (*p* < 0.001). Evaluators consistently perceived 3DPT as more similar to NT than AT. Regarding treatment outcomes, 3DPT showed significantly higher scores for endodontic preparation, verifier fitting, and root canal filling (*p* < 0.05), while presenting significantly fewer procedural errors than AT (*p* < 0.001). **Conclusions:** PolyJet™ 3DPT fabricated with RGD525™ demonstrated promising applicability for preclinical endodontic training, combining favorable perceptions, fewer procedural errors, and potential for low-cost large-scale in-house production. Nevertheless, improvements in material realism and tactile simulation are still required.

## 1. Introduction

Endodontics constitutes a fundamental area within dentistry, focusing on the identification, prevention, and management of conditions involving the dental pulp and surrounding periapical tissues. The outcome of endodontic therapy is influenced by multiple procedural steps, particularly adequate canal shaping, effective disinfection, and proper three-dimensional obturation [1]. The European Society of Endodontology has established recommendations for dental schools regarding the organization of endodontic teaching, highlighting the importance of integrating both preclinical training and clinical experience [2]. Within academic environments, especially during the initial stages of learning, preclinical training plays a crucial role by equipping students with the competencies required to perform treatments in a safe and efficient manner when managing patients [1,3]. Key factors supporting the adoption of simulation artificial models in dental education include the provision of a safe learning environment, equal training opportunities for all students, and an easier transition from preclinical teaching to patient care through artificial models that approximate clinical conditions. Simulation-based training also promotes efficient learning, broad access to practice resources, objective feedback, flexible training schedules, and greater cost-efficiency for educational institutions [4,5].

Preclinical endodontic instruction has traditionally relied on extracted human teeth. Their use provides students with direct contact with authentic root canal morphology and the inherent mechanical properties of dentin. However, incorporating natural teeth (NT) into teaching settings requires strict adherence to infection prevention protocols to minimize the risk of cross-contamination, in addition, ethically sourced teeth obtained with informed consent are becoming progressively harder to secure [6,7]. Moreover, anatomical variability among NT may hinder the assessment of learning progress and limit the development of students’ self-evaluation skills. Another relevant issue is that preclinical endodontic grading often relies on subjective teacher judgment, which may reduce the quality of feedback, compromise scoring fairness, and generate student dissatisfaction [8]. Consequently, selecting comparable NT specimens is labor-intensive and requires access to an extensive tooth bank; nevertheless, this is often not feasible [6,7].

Commercial artificial teeth (AT) have been introduced to address these limitations by providing specimens with uniform complexity and broad availability for student training. Their use in preclinical education has increasingly expanded, being recognized as a practical alternative for endodontic instruction. Currently, a range of AT exists, offering standardized configurations with varying degrees of anatomical realism [9]. Despite their advantages, AT are often linked to elevated costs, a restricted variety of tooth anatomies, and prolonged delivery periods resulting from dependence on external manufacturers. Furthermore, commercial brands differ considerably in their manufacturing methods and materials [7,8,9].

Three-dimensional (3D) printing has rapidly evolved and is now widely integrated into dentistry. Advances in affordability and material availability have made 3D-printed teeth (3DPT) a viable option for producing customized artificial models that may not be commercially available or economically feasible in large quantities [7,8].

Surveys evaluating undergraduate endodontic education in different countries have shown considerable variability in the preclinical training resources used [1,3,10,11,12,13]. NT remain the most used training model worldwide; however, many dental schools also incorporate AT, acrylic resin blocks, and, more recently, 3DPT into their curricula. Studies from the United Kingdom, Canada, Italy, and Spain reported heterogeneous adoption of these artificial models, with increasing integration of 3DPT in preclinical teaching [10,11,12,13]. Similarly, an international survey involving dental schools from 36 countries reported considerable variability in preclinical endodontic training approaches, with some institutions exclusively using NT or AT, whereas others adopted a hybrid approach combining NT, AT, and 3DPT, the latter already being integrated into the curricula of 25.1% of the evaluated schools [1].

Previous studies on students’ perceptions of 3DPT consistently indicate that the main limitations remain in reduced hardness/tactile realism and suboptimal radiopacity; nevertheless, despite these recurring concerns, students generally report good acceptance and positive attitudes toward their use in preclinical endodontic training [9]. Multiple printing technologies and material options are available, each presenting specific strengths and limitations, making appropriate material selection a critical factor [14].

Our previous studies extensively evaluated the limitations and performance of PolyJet™ 3DPT, establishing their suitability for endodontic research applications [15,16,17]. In PolyJet™ printing (Stratasys Ltd., Eden Prairie, MN, USA), multiple nozzles move across the XY plane, depositing a liquid photopolymer that is immediately cured by ultraviolet light, while the build platform descends layer by layer along the Z-axis until completion and allows for the simultaneous deposition of removable support material for complex hollow or overhanging structures, making it particularly suitable for medical prototyping. The material used in our studies, RGD525™ (Stratasys Ltd., Eden Prairie, MN, USA), exhibits mechanical properties comparable to several relevant parameters of human dentin, including tensile strength and elastic modulus, despite differences in internal microstructure [15,16,17]. Therefore, these 3DPT may also represent a promising alternative for preclinical endodontic education, warranting the evaluation of students’ and evaluators’ perceptions regarding their realism and handling characteristics.

The present study aimed to evaluate the perceptions of undergraduate students with no previous experience using AT or 3DPT regarding these two artificial models. Students’ perceptions were assessed by comparing AT and 3DPT with each other and by using NT only as a perceptual and educational reference, reflecting the preclinical standard familiar to students and evaluators. NT were therefore not included as a procedural experimental group. Additionally, evaluators’ perceptions of AT and 3DPT were assessed to determine whether these artificial models could be considered suitable substitutes or complements to NT for teaching purposes. Finally, the study compared the quality of endodontic treatments performed by undergraduate students using AT and 3DPT, in order to determine whether the type of artificial training model influenced the procedural outcomes.

## 2. Materials and Methods

The study was conducted in accordance with the Declaration of Helsinki. Ethical approval for the participation of students was obtained from the Ethics Committee of the University of Santiago de Compostela (USC-009/2026-H, 25 February 2026). Ethical approval for the creation of the micro-CT tooth library used for specimen selection was previously granted by the Ethics Committee of Fernando Pessoa University (FCS/PI 636/24, 10 December 2024).

The study involved five stages: (1) questionnaire development and validation; (2) tooth selection (3) PolyJet™ 3DPT fabrication; (4) experimental sessions; and (5) artificial models’ evaluation by undergraduate students and evaluators using the developed questionnaires.

The study sample comprised undergraduate dental students from the University of Santiago de Compostela. Eligible participants were students attending the Dental Pathology and Therapeutics II Unit during the fourth year, ensuring one semester previous of preclinical experience in endodontics using NT. From the pool of eligible students, none had previous experience performing preclinical procedures using AT or 3DPT; therefore, no participants were excluded.

All 47 eligible students voluntarily agreed to participate in the study. Written informed consent was obtained from each participant before the beginning of the study. Participation was optional, and the decision to participate or decline had no effect on academic progression. Questionnaire responses and treatment outcomes were anonymized and could not be linked to individual performance. All data were processed and stored in accordance with applicable data protection regulations.

The panel of evaluators comprised three faculty members with teaching experience in endodontics and previous experience assessing student treatments. None had prior experience of grading procedures performed on AT or 3DPT.

### 2.1. Stage 1—Questionnaire Development and Validation

In line with the methodological approach adopted in previous studies assessing students’ perceptions of AT and 3DPT in preclinical endodontic training, the questionnaire used NT as the reference model for comparison [18,19,20]. The questionnaire applied in this study was adapted from a previously published instrument [9], which was subsequently modified and translated into Spanish to suit the objectives of the present investigation (Table S1). The revision process involved four authors with clinical and academic experience. The main purpose of these modifications was to ensure that the instrument adequately addressed the specific outcomes under evaluation. Particular attention was also given to improving the clarity and accessibility of each item. Technical terminology and wording were carefully adjusted so that the content would be readily understood by dental students. After revision, the final version was examined by all members of the team to confirm coherence, comprehensibility, and consistency with the aims of the study.

The questionnaire consisted of structured items using a 5-point Likert scale. Students were asked to compare both AT and 3DPT regarding external and internal anatomy, pulp chamber and root canal morphology, radiopacity, visibility of instruments and verifiers on radiographs, tactile sensation during access cavity preparation and instrumentation, and debris removal during irrigation, in comparison with NT. Additional items evaluated the perceived suitability of artificial models for understanding nonsurgical endodontic treatment, practical classes, continuous assessment, practical examinations, hygiene, and overall preference for use in preclinical endodontic training. Evaluators completed an adapted version of the same questionnaire, excluding items related to tactile perception and the direct procedural experience of endodontic treatment.

A pilot pretest with the target population was not feasible prior to data collection, as meaningful evaluation of the questionnaire items required prior hands-on experience with the tested endodontic artificial models. Therefore, content validity was established through expert review, and reliability was subsequently assessed through a test–retest design after participants completed the practical sessions. Twenty students participating in the first experimental session completed the questionnaire again one week after the initial administration, and concordance between responses was analyzed to evaluate temporal stability of the instrument. To enable pairing of questionnaire responses collected at different time points while preserving anonymity, participants were asked to generate a personal alphanumeric code known only to themselves. The code was created using predefined elements: the first two letters of their favorite animal, the numerical day of birth (01–31), the last two letters of their mother’s first name, and the last two letters of their father’s first name. All entries were recorded in lowercase letters and numbers only. This procedure allowed for the longitudinal matching of responses for validation purposes without collecting directly identifiable personal information.

A separate evaluator scoring form was used to assess the technical quality of the treatments performed on each tooth. For every specimen, evaluators independently rated the main treatment stages using a 5-point scale. The evaluated domains included access cavity preparation, working length determination, endodontic preparation, verifier adaptation, and root canal obturation. In addition, evaluators registered the presence or absence of procedural errors. For access cavity preparation, excessive removal of tooth structure and perforations were recorded. During instrumentation, errors included overpreparation, under preparation, ledge formation, debris blockage, canal perforation, apical transportation, and instrument fracture. For obturation, overfilling, underfilling, and the presence of voids were also documented.

### 2.2. Stage 2—Tooth Selection

From an existing micro-CT library of NT, created by scanning NT with a micro-CT system (Skyscan 1174; Bruker, Kontich, Belgium) at 50 kV and 800 μA, using a 0.25 mm aluminium filter, 0.9° rotational steps over 180°, a voxel size of 19.60 μm, and an exposure time of 12,000 ms, a mandibular premolar was selected for 3D printing.

**Figure 1:**
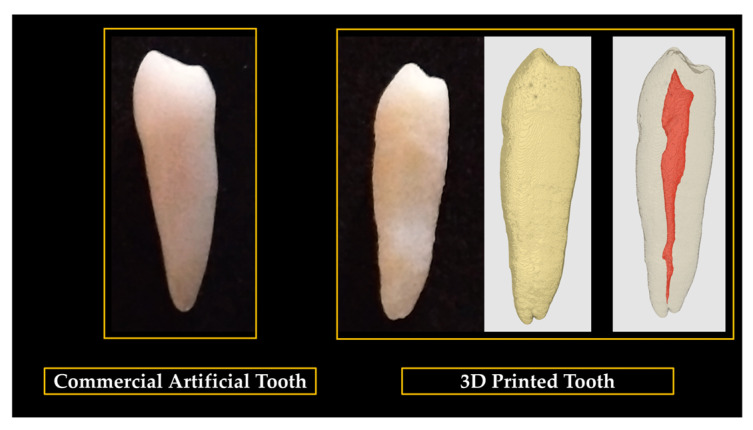
Representative images of external appearance of the commercial artificial tooth and the 3D-printed tooth used in the present study. The 3D-printed tooth additionally includes the corresponding three-dimensional reconstructions of the external anatomy and root canal system obtained from the STL design used for printing.

A corresponding mandibular premolar with a white crown and root from DRSK Group DRSK Endodontic™ (DRSK Group, Hassleholm, Sweden; tooth code: 3411-101-RRO), described by the manufacturer as featuring simulated pulp, dentin-like tactile sensation, and radiographic contrast [21], was selected for comparison (Figure 1).

Premolars were chosen because of their relatively simple root canal anatomy, making them suitable for undergraduate students with limited previous experience in preclinical endodontics. In addition, the use of a less complex tooth morphology was considered appropriate given the restricted duration of the experimental sessions, allowing participants to complete all stages of endodontic treatment in both artificial models within the available time.

### 2.3. Stage 3—PolyJet™ 3DPT Fabrication

From the micro-CT dataset, image reconstruction was performed with NRecon software (version 1.7.46; Bruker, Kontich, Belgium) using ring artifact correction (3), smoothing (3), and beam hardening correction (40%), and the reconstructed datasets were processed in CTAn software (version 1.20.3.0; Bruker, Kontich, Belgium) to generate a STL file of the selected mandibular premolar. The STL model was then manufactured using PolyJet™ technology with a Stratasys Objet30 Prime™ printer (Stratasys Ltd., Eden Prairie, MN, USA) in High Quality mode, with a layer thickness of 16 μm. RGD525™ high-temperature resin and SUP706B™ support material (Stratasys Ltd., Eden Prairie, MN, USA) were used for fabrication. Based on our previous studies [15,17], the tooth was positioned on the build platform with the root long axis parallel to the Y axis. In addition, the major root cross-sectional axis and the major canal cross-sectional axis were both oriented parallel to the build platform to optimize printing accuracy. All 3DPT used in the present study were produced in a single batch three months before the experimental sessions and stored in an opaque closed container, protected from direct sunlight and under seasonal temperature variation (5–20 °C) (Figure 1).

### 2.4. Stage 4—Experimental Sessions

This stage consisted of two experimental sessions separated by a one-week interval. Twenty-five students participated in the first session and twenty-two in the second. The sessions were divided in this manner because the students’ academic schedule did not permit all participants to be accommodated in a single session. Two weeks before the experimental sessions, a theoretical session was provided to all 47 participants to explain the study protocol and objectives. During this session, students were instructed that all stages of nonsurgical endodontic treatment had to be performed in parallel on both teeth. Accordingly, each procedural step was completed on both teeth before progressing to the next stage. The treatment protocol and materials used during the study were identical to those routinely employed in the participants’ preclinical training. In addition, all experimental sessions were conducted in the same laboratory facilities where their regular practical classes take place. The Endogal^®^ system (Endogal, Galician Endodontics Company, Sarria, Lugo, Spain) was used for root canal preparation, followed by obturation with Endogal^®^ obturators (Endogal, Galician Endodontics Company, Sarria, Lugo, Spain), using Endogal^®^ Endoresin cement (Endogal, Galician Endodontics Company, Sarria, Lugo, Spain). Radiographic procedures were performed by the students using the same X-ray unit employed in preclinical training Carestream™ CS 2200 Intraoral X-Ray Unit (Carestream Dental LLC, Atlanta, GA, USA), together with a Carestream™ 6100 RVG digital imaging system (Carestream Dental LLC, Atlanta, GA, USA). Radiographs were obtained in the mesiodistal direction to improve the visualization and identification of procedural errors.

The teeth were randomly distributed among participants. To ensure anonymity, each pair of teeth was assigned an FDI tooth number code that was visible only to the respective student, preventing any linkage between the participant’s identity and the treated artificial models. All radiographs were saved by the students in the Carestream™ software (CS Imaging Version 7) under the assigned code, allowing the evaluators to later evaluate the completed treatments. For data organization, two separate virtual “patients” were created in the software, one corresponding to each experimental session.

### 2.5. Stage 5—Artificial Models Evaluation

At the end of each experimental session, a QR code was provided to the participants. By scanning the code, students were directed to a Google Forms questionnaire designed to collect their perceptions regarding the realism, handling characteristics, and educational usefulness of the evaluated teeth.

**Figure 2:**
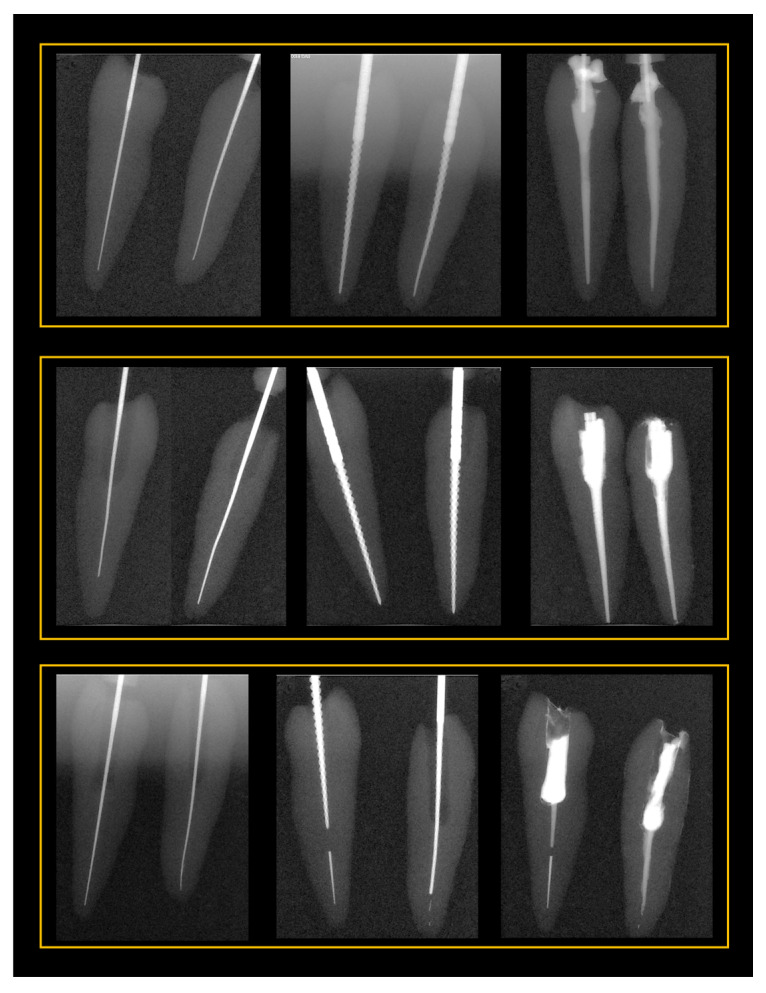
Representative radiographs of endodontic procedures performed by undergraduate students in commercial artificial teeth and 3D-printed teeth, selected to illustrate the variability of procedural outcomes observed during preclinical endodontic training. The upper rows show more acceptable procedural outcomes, whereas the lower row illustrates less favorable outcomes. Each row shows one student case and includes the radiographs used for evaluator assessment. From left to right, the images show working length determination, verifier fitting, and root canal filling. In all images, commercial artificial teeth is positioned on the left and 3D-printed teeth on the right.

Similarly, the evaluators completed a Google Forms questionnaire regarding their perceptions of the tested artificial models, as well as a separate Google Forms form for the assessment of each treated pair of teeth. For this purpose, a Microsoft PowerPoint (Microsoft 365, Microsoft Corp., Redmond, WA, USA) presentation was prepared, in which each slide corresponded to one pair of teeth and included all radiographs obtained throughout treatment for that pair. In addition, the physical teeth remained available for direct visual inspection during the evaluation process (Figure 2).

To facilitate interpretation of the overall perceptions of both students and evaluators, Likert-scale responses were regrouped into three categories for each comparison performed (AT vs. NT and 3DPT vs. NT). Scores of 1 and 2 were classified as negative opinions in comparison with NT, scores of 4 and 5 as positive opinions in comparison with NT, and a score of 3 as a neutral opinion, indicating equivalence with NT for that specific item [8,9,22].

### 2.6. Statistical Analysis

Statistical analysis was performed using IBM SPSS Statistics version 30.0 (IBM Corp., Armonk, NY, USA). The level of significance was set at α = 0.05. Descriptive statistics were used to summarize the data. Continuous and ordinal variables were expressed as mean ± standard deviation (SD) and median with interquartile range (IQR), while categorical variables were presented as frequencies and percentages. Data normality was assessed using the Shapiro–Wilk test. As most variables were not normally distributed, non-parametric tests were applied. Comparisons between AT and 3DPT were performed using the Wilcoxon signed-rank test for paired samples. Comparisons among the three related perception ratings—NT, AT, and 3DPT—were performed using the Friedman test, followed by Wilcoxon signed-rank post hoc tests when appropriate. Inter-examiner reliability for quantitative assessments was evaluated using the intraclass correlation coefficient (ICC), based on a two-way mixed-effects model with absolute agreement. Agreement in the identification of categorical variables, namely procedural errors, was assessed using Fleiss’ kappa coefficient. Test–retest reliability of the questionnaire was evaluated using weighted Cohen’s kappa. Due to the small number of expert evaluators (*n* = 3), their assessments were analyzed descriptively, without inferential statistical testing.

## 3. Results

**Figure 3:**
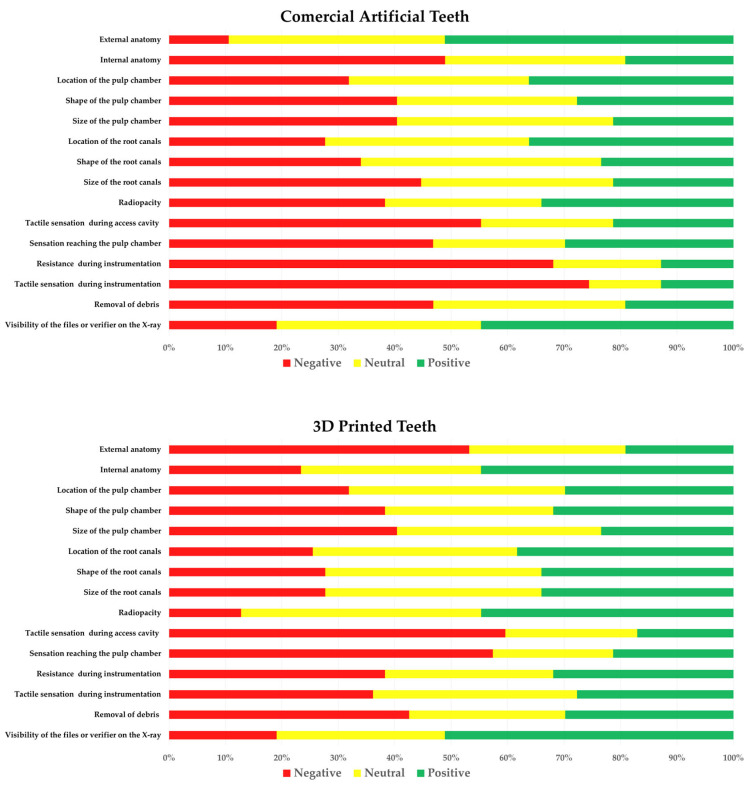
Distribution of students’ perceptions regarding the similarity of commercial artificial teeth and 3D-printed teeth in comparison to natural teeth, grouped into negative (scores 1–2), neutral (score 3), and positive (scores 4–5) responses.

Students’ perceptions regarding the similarity of AT and 3DPT in comparison to NT are presented in Table 1 and summarized graphically in Figure 3, whereas direct comparisons between both artificial models are shown in Table 2. AT demonstrated greater perceived similarity to NT for external anatomy. In contrast, procedural and tactile parameters, particularly tactile sensation during access cavity preparation, resistance of root canal walls and tactile sensation during instrumentation, received predominantly negative ratings (all *p* < 0.001). Perceptions of 3DPT were generally more balanced across anatomical parameters and showed more favorable ratings for radiopacity and radiographic visibility (*p* = 0.007). Direct comparisons between the artificial models revealed significantly higher ratings for AT in external anatomy, whereas 3DPT received significantly higher ratings for internal anatomy, radiopacity, resistance of root canal walls and tactile sensation during instrumentation (*p* = 0.002). No significant differences were identified for the remaining parameters.

Weighted Cohen’s kappa demonstrated good to excellent test–retest reliability across all questionnaire items, with values ranging from 0.685 to 0.937, supporting the reliability of the instrument (Table S2).

When AT and 3DPT were analyzed together as artificial models (Table 3), no significant differences in comparison with NT were observed regarding educational applicability, including understanding non-surgical endodontic treatment, practical classes, continuous assessment, and practical examinations (*p* > 0.05). Artificial models were, however, perceived as significantly more hygienic than NT (*p* < 0.001). Students’ preference rankings are presented in Table 4. NT received the highest preference scores and were significantly preferred over both AT and 3DPT (*p* < 0.001). Additionally, 3DPT were significantly preferred over AT (*p* = 0.005).

Evaluators’ perceptions regarding the similarity between artificial models in comparison to NT are presented in Table 5. The 3DPT were consistently perceived as more similar to NT than AT, particularly regarding internal anatomy, pulp chamber characteristics, and root canal morphology. Evaluators’ perceptions regarding the educational applicability of artificial models are summarized in Table 6. Positive ratings were observed mainly for continuous assessment, practical examinations, and hygiene. Preference rankings assigned by evaluators are presented in Table 7, in which NT and 3DPT received higher ratings than AT.

Inter-examiner reliability demonstrated excellent agreement across all evaluated procedures, with ICC values ranging from 0.947 to 0.976 (Table S3). Table 8 presents the evaluators’ assessment of students’ endodontic treatment outcomes performed using AT and 3DPT. Comparable scores were observed for access cavity preparation and working length determination. In contrast, 3DPT achieved significantly higher scores for endodontic preparation, verifier fitting, and root canal filling (*p* < 0.001). Agreement for procedural error identification demonstrated excellent consistency among evaluators, with Fleiss’ kappa values ranging from 0.889 to 1.000 (Table S4). The distribution of procedural errors according to tooth type is presented in Table 9. Higher frequencies of errors were consistently observed in AT, particularly for underfilling, under-preparation, debris blockages, instrument fracture, and obturation voids. Excessive removal of tooth structure during access cavity preparation was also more frequent in AT. Ledge formation, canal perforation, and apical transportation showed low occurrence in both groups. The total number of procedural errors per tooth is presented in Table 10. AT showed a significantly higher number of errors per tooth compared with 3DPT (*p* < 0.001).

## 4. Discussion

The present study investigated the perceptions of students and evaluators with no previous experience using artificial models regarding AT and PolyJet™ 3DPT fabricated with RGD525™ for preclinical endodontic training, while simultaneously assessing the quality of the endodontic procedures performed using both artificial models. The number of participating students was limited by the size of the dental program and by the need to include participants with an adequate and homogeneous baseline level of theoretical and practical endodontic knowledge. Therefore, all students were recruited from the same stage of training and had received comparable preclinical instruction, ensuring a similar educational background for the comparisons performed [23].

The test–retest analysis of the student questionnaire confirmed good to excellent reliability (weighted Cohen’s kappa values from 0.685 to 0.937) across all evaluated items, supporting the reliability and consistency of questionnaire responses.

Regarding students’ perceptions, AT received more favorable evaluations for external anatomy classified as positive (*p* < 0.05). In contrast, procedural and tactile aspects were predominantly negatively rated, particularly tactile sensation during access cavity preparation, resistance of the root canal walls and tactile sensation during instrumentation (all *p* < 0.001), suggesting lower perceived realism during operative procedures.

For 3DPT, most anatomical parameters showed balanced perceptions without significant deviations from neutrality (*p* > 0.05). External anatomy was the only anatomical characteristic associated with predominantly negative responses (*p* < 0.05). Conversely, positive perceptions were identified for radiopacity, sensation of reaching the pulp chamber, and radiographic visibility (all *p* < 0.05), with radiographic-related variables generally receiving median scores between 3 and 4.

Direct comparisons between both artificial models revealed significant differences for several parameters. AT received higher ratings for external anatomy (*p* < 0.001), whereas 3DPT were more positively evaluated for internal anatomy and radiopacity (both *p* < 0.001). Instrumentation-related variables also favored 3DPT, which showed significantly higher ratings for resistance of the root canal walls (*p* < 0.05) and tactile sensation of the files during instrumentation (*p* < 0.001). The remaining evaluated parameters showed no significant differences between artificial models (*p* > 0.05).

When AT and 3DPT were analyzed together as artificial models, students did not identify significant differences in comparison with NT regarding most educational applications, including understanding non-surgical endodontic treatment, practical classes, continuous assessment, and practical examinations (*p* > 0.05). In contrast, artificial models were perceived as significantly more hygienic than NT, with 89.4% of responses classified as positive (*p* < 0.001). Despite these favorable educational perceptions, NT remained the preferred training model, followed by 3DPT, whereas AT received the lowest preference ratings (*p* < 0.001).

A previous study evaluating the perceptions of 220 dental students with experience using both AT, specifically DRSK models and NT, reported positive perceptions regarding external anatomy, hygiene, availability, and ease of handling, whereas negative perceptions were identified for internal anatomy, pulp chamber characteristics, canal morphology, radiopacity, tactile feedback, debris removal, and suitability for understanding endodontic procedures [9]. The findings of the present study are partially consistent with these observations. Similarly to the previous investigation, AT received favorable evaluations for external anatomy, while tactile and procedural aspects were predominantly negatively rated. Artificial models were also perceived as more hygienic than NT. However, unlike the previous report, the present study did not demonstrate a generally negative perception regarding the educational applicability of artificial models. This discrepancy should be interpreted cautiously, since the previous investigation evaluated only AT versus NT, whereas the present study assessed AT and 3DPT together. Therefore, the more favorable educational perceptions observed may have been influenced by the inclusion of 3DPT, which received significantly higher ratings than AT for internal anatomy, radiopacity, root canal wall resistance, and tactile sensation during instrumentation. These findings suggest that, although AT still present limitations regarding tactile feedback and procedural simulation, 3DPT may overcome some of these shortcomings and improve the perception of artificial models for preclinical endodontic training. Previous studies evaluating the reproduction of pulp anatomy in 3DPT have reported inconsistent findings. While some investigations described pulp replication as only satisfactory or even unsatisfactory in reproducing natural conditions [18], others reported correct pulp morphology and identifiable anatomical references for conventional access cavity preparation [20]. The findings of the present study are more consistent with the latter observations, as the evaluated 3DPT received predominantly positive perceptions regarding pulp chamber location, shape, and size, suggesting a favorable reproduction of internal anatomical characteristics.

Limitations related to hardness, tactile feedback, and radiopacity have also been frequently described in previous studies evaluating 3DPT for endodontic education, with students commonly perceiving 3DPT as softer and less realistic than NT during instrumentation procedures [4,8,18,19,20,23,24]. Similar limitations were still observed in the present investigation, particularly regarding tactile sensation during access cavity preparation. Nevertheless, more favorable findings were identified for instrumentation-related parameters. Students did not perceive 3DPT as significantly different from NT regarding resistance of the root canal walls or tactile sensation of the files during instrumentation, and both variables received significantly higher ratings for 3DPT than for AT. These findings suggest that the PolyJet™ 3DPT fabricated with RGD525™ may provide a more realistic instrumentation experience than previously reported for other 3DPT. Likewise, radiopacity and radiographic visibility were positively perceived, contrasting with earlier investigations in which low radiopacity was considered a major limitation of 3DPT [4,8,18,19,20,23,24]. Such differences may be associated with variations in printing technologies, materials, and model design.

From the evaluators’ perspective, 3DPT were consistently perceived as more similar to NT than AT across most evaluated parameters. In contrast, AT received lower evaluations for most anatomical characteristics, suggesting a lower perceived similarity to natural structures. Radiopacity received comparatively lower ratings for both artificial models, although 3DPT were still slightly better evaluated than AT. Together, these findings suggest that 3DPT may provide a more realistic anatomical simulation than AT, while maintaining comparable radiographic visualization during endodontic procedures.

Consistent with the previous literature [4,22], evaluators in the present study positively rated artificial models across all assessed domains. The highest evaluations were observed for continuous assessment, practical examinations, and hygiene, with complete agreement among evaluators. Artificial models were also considered suitable for understanding non-surgical endodontic treatment and for practical endodontic classes, although with slightly greater variability in responses. Preference rankings further reinforced these perceptions, with both NT and 3DPT receiving the highest scores among evaluators, whereas AT were consistently less preferred and demonstrated greater variability in ratings.

Differences between students’ and evaluators’ perceptions observed in the present study may be related to their distinct clinical and teaching experience. Evaluators appeared to place greater emphasis on the advantages of artificial models regarding standardization, hygiene, availability, and assessment consistency, likely reflecting their broader experience with the limitations associated with preclinical training using NT. In contrast, students tended to be more critical regarding tactile sensation and instrumentation-related characteristics, which directly influence procedural execution [6].

The evaluation methodology demonstrated high reproducibility, with excellent inter-examiner agreement observed for both treatment quality assessment and procedural error identification. ICC values ranged from 0.947 to 0.976, while Fleiss’ kappa values ranged from 0.889 to 1.000, supporting the reliability and consistency of the evaluators’ assessments.

When treatment outcomes were analyzed, no statistically significant differences were identified between AT and 3DPT for access cavity preparation or working length determination (*p* > 0.05), suggesting comparable performance during the initial stages of treatment. However, 3DPT received significantly higher scores for endodontic preparation, verifier fitting, and root canal filling (all *p* < 0.001), indicating superior performance during instrumentation and obturation procedures.

Procedural error analysis further reinforced these findings, as AT showed a consistently higher frequency of errors across most evaluated parameters. The most common errors observed in AT were underfilling, under-preparation, and debris blockages, followed by instrument fracture and obturation voids. In contrast, these errors occurred substantially less frequently in 3DPT. Excessive removal of tooth structure during access cavity preparation was also more common in AT, whereas ledge formation, canal perforation, and apical transportation showed low frequencies in both groups.

The analysis of total procedural errors per tooth further reinforced the differences observed between the evaluated artificial models. AT showed a significantly higher number of errors per tooth than 3DPT (*p* < 0.001), with a mean of 3.53 ± 1.74 errors and a median of 4 (2–5), whereas 3DPT presented a mean of 1.49 ± 1.44 errors and a median of 1 (0–2). These findings indicate a substantially greater procedural error burden associated with AT.

Similar observations have been reported in previous studies describing reduced tactile sensation, debris accumulation, and increased risk of procedural errors during instrumentation of AT [22,25,26,27]. In the present study, the high frequency of debris blockages observed in AT may be associated with the physical properties of the materials used in their manufacture, particularly the production of adhesive debris capable of obstructing the canals and impairing instrumentation procedures. This phenomenon may also help explain the higher frequencies of under-preparation, instrument fracture, and underfilling identified in AT.

A previous investigation additionally suggested that the hardness of AT materials may become particularly relevant when rotary instrumentation and warm obturation techniques are used, as performed in the present study, whereas such limitations may be less pronounced during manual instrumentation and cold lateral compaction procedures [28]. This may further justify the higher frequency of instrumentation-related procedural errors observed in AT.

Another aspect that should be considered is the lack of previous experience of the participating students with either AT or 3DPT. Consequently, part of the procedural errors identified may also reflect the initial adaptation required for handling artificial models, as previous studies have reported that students performing these procedures for the first time tend to commit a high errors percentage [29]. Nevertheless, despite this absence of prior experience, 3DPT still demonstrated substantially fewer procedural errors than AT, suggesting that their material properties and instrumentation behavior may provide a more favorable operative experience during preclinical endodontic training.

The cost of tooth replicas varies according to the selected tooth type and the level of anatomical detail reproduced. Since preclinical endodontic training requires the repeated use of multiple specimens by large numbers of students simultaneously, affordability represents an important characteristic for any educational model. In addition, the availability of lower-cost training resources may help reduce part of the financial burden associated with dental education, which has been recognized as a relevant source of stress among dental students [14]. In the present study, based on the market prices in the country where the investigation was conducted, the material cost of each 3DPT corresponded to only 4.2% of the cost of the AT used for comparison, with AT being approximately 24 times more expensive than 3DPT. Although this estimation did not include printer acquisition, maintenance, or technical operation costs, these findings support the potential economic viability of large-scale in-house production of 3DPT for dental education, particularly considering that minimum preclinical training requirements in some dental schools may reach up to 30 teeth per student [1].

A notable methodological strength of the present study is the combined assessment of students’ perceptions, questionnaire reliability, allowing a broader evaluation of the educational value and practical performance of the tested training models [30]. The questionnaire was adapted to the specific objectives of the study and subjected to a test–retest reliability assessment, strengthening the consistency of the perception-based data. Also, by integrating perception data with objective procedural assessment, this approach provides a more comprehensive understanding of the potential role of AT and 3DPT in preclinical endodontic education.

Our study presents several limitations, similarly to previous investigations evaluating 3DPT for endodontic education [30,31]. Although the high degree of protocol standardization strengthened internal validity, it may also reduce generalizability. First, the study was conducted using a single tooth morphology, namely mandibular premolars with relatively simple root canal anatomy. Therefore, the findings may not be directly extrapolated to teeth with more complex anatomies, such as molars, curved canals, multiple canals, or teeth with anatomical variations. Second, all procedures were performed under controlled conditions using standardized instrumentation and obturation protocols, which may not fully reflect the variability of university teaching environments. Future studies should include different tooth types, more complex canal anatomies, and broader procedural conditions to better determine the educational applicability of PolyJet™ 3DPT across different preclinical scenarios. In addition, the intervention period was limited to a single practical class rather than a conventional preclinical endodontic course extending over several weeks. Consequently, the present findings mainly reflect initial-stage responses and early differences between the training modalities evaluated. Repeated practice combined with continuous feedback over longer periods could potentially modify the observed differences and further improve students’ abilities. Therefore, future studies involving students with no previous experience using artificial models and longer training periods are warranted to better evaluate adaptation over time, the learning curve associated with these artificial models, and their educational impact throughout preclinical endodontic training.

In the present study, students were allowed to choose the order in which they performed the procedures on the AT and the 3DPT. Although they were instructed to complete each procedural step sequentially in both models, the individual order selected by each participant was not recorded. Therefore, a possible order or practice effect cannot be excluded, since performance on the second model may have been influenced by the experience gained during the first [30]. In addition, allowing participants to choose the order may have introduced a preference-related bias, as some students may have started with the model, they perceived as easier or more familiar. In this way, future studies should record and randomize the order of model use, preferably using a counterbalanced design, to allow a more robust comparison between AT and 3DPT [30].

Another limitation relates to the experimental setup itself. Although the tested teeth proved adequate for the purposes of the present investigation, they were evaluated only as individual units handled directly by students. Despite the widespread use of tooth replicas in dental education, modular 3D-printed systems capable of simulating different clinical scenarios remain limited. The development of customized training systems adapted to specific curricular requirements may represent an important advantage for dental schools, provided that the manufacturing workflow is feasible and the simulations achieve realistic performance [24]. In this context, future integration of 3DPT into modular simulation bases or clinical training setups could increase the versatility, realism, and educational applicability of these systems. Our previous studies demonstrated that PolyJet™ 3DPT fabricated with RGD525™ may be successfully used in endodontic research applications [15,16,17]. In the present study, the lower ratings observed for external anatomy may be partially explained by the fact that this material is currently available only in white, limiting the esthetic reproduction of enamel and dentin structures. Nevertheless, students reported more favorable perceptions regarding instrumentation-related tactile sensation in PolyJet™ 3DPT fabricated with RGD525™, suggesting that the material may provide an adequate simulation of dentin behavior during canal preparation. In contrast, tactile sensation during access cavity preparation remained less favorably perceived and similar to AT, indicating that the reproduction of enamel characteristics may still represent a limitation. Previous studies have already described the development of reinforced additively manufactured composites and filler compounds for radiopaque PolyJet™ multi-material applications, suggesting that such approaches may represent promising strategies to improve the mechanical, tactile, and radiographic properties of PolyJet™ 3DPT [32,33]. Consequently, future developments in printing materials, including dentin- and enamel-like color variations, as well as modified materials with fillers capable of better simulating enamel properties, may contribute to improved visual realism and tactile performance of PolyJet™ 3DPT fabricated with RGD525™.

## 5. Conclusions

Within the limitations of the present study, PolyJet™ 3DPT fabricated with RGD525™ demonstrated promising applicability for preclinical endodontic training. Compared with AT, these 3DPT provided more favorable perceptions regarding internal anatomy, radiopacity, and instrumentation-related tactile sensation, while also showing significantly fewer procedural errors during endodontic procedures. In addition, their substantially lower production cost and potential for large-scale in-house manufacturing support their feasibility as a standardized and economically viable alternative for undergraduate endodontic education. Nevertheless, further developments in material properties, visual realism, and tactile performance are still required to achieve a more biomimetic and haptic simulation of NT.

## Figures and Tables

**Table 1:** Students’ perceptions of commercial artificial teeth and 3D-printed teeth in comparison with natural teeth.

	Commercial Artificial Teeth								3D-Printed Teeth							
	Negative		Neutral		Positive		Median (IQR)	*p*	Negative		Neutral		Positive		Median (IQR)	*p*
	n	%	n	%	n	%			n	%	n	%	n	%		
External anatomy	5	10.6	18	38.3	24	51.1	4 (3–4)	**0.002**	25	53.2	13	27.7	9	19.1	2 (2–3)	**0.012**
Internal anatomy	23	48.9	15	31.9	9	19.1	3 (2–3)	**0.008**	11	23.4	15	31.9	21	44.7	3 (3–4)	0.115
Location of the pulp chamber	15	31.9	15	31.9	17	36.2	3 (2–4)	0.937	15	31.9	18	38.3	14	29.8	3 (2–4)	0.890
Shape of the pulp chamber	19	40.4	15	31.9	13	27.7	3 (2–4)	0.097	18	38.3	14	29.8	15	31.9	3 (2–4)	0.496
Size of the pulp chamber	19	40.4	18	38.3	10	21.3	3 (2–3)	0.056	19	40.4	17	36.2	11	23.4	3 (2–3)	0.186
Location of the root canals	13	27.7	17	36.2	17	36.2	3 (2–4)	0.282	12	25.5	17	36.2	18	38.3	3 (2–4)	0.249
Shape of the root canals	16	34.0	20	42.6	11	23.4	3 (2–3)	0.295	13	27.7	18	38.3	16	34.0	3 (2–4)	0.575
Size of the root canals	21	44.7	16	34.0	10	21.3	3 (2–3)	0.160	13	27.7	18	38.3	16	34.0	3 (2–4)	0.964
Radiopacity	18	38.3	13	27.7	16	34.0	3 (2–4)	0.936	6	12.8	20	42.6	21	44.7	3 (3–4)	**0.003**
The tactile sensation produced by the turbine and bur during access cavity preparation	26	55.3	11	23.4	10	21.3	2 (1–3)	**<0.001**	28	59.6	11	23.4	8	17.0	2 (1–3)	**<0.001**
The sensation of sudden drop upon reaching the pulp chamber	22	46.8	11	23.4	14	29.8	3 (2–4)	0.307	27	57.4	10	21.3	10	21.3	2 (1–3)	**0.002**
The resistance of the root canal walls during instrumentation	32	68.1	9	19.1	6	12.8	2 (1–3)	**<0.001**	18	38.3	14	29.8	15	31.9	3 (2–4)	0.138
The tactile sensation of the files during instrumentation	35	74.5	6	12.8	6	12.8	2 (2–3)	**<0.001**	17	36.2	17	36.2	13	27.7	3 (2–4)	0.251
The removal of debris formed during instrumentation, using irrigation.	22	46.8	16	34.0	9	19.1	3 (2–3)	**0.024**	20	42.6	13	27.7	14	29.8	3 (2–4)	0.097
Visibility of the files or verifier on the X-ray	9	19.1	17	36.2	21	44.7	3 (3–5)	**0.001**	9	19.1	14	29.8	24	51.1	4 (3–5)	**0.007**

IQR—interquartile range. Values are presented as frequencies (n, %) and median (IQR). Negative responses correspond to scores 1–2, neutral responses to score 3, and positive responses to scores 4–5. *p*-values correspond to comparisons against a neutral distribution.

**Table 2:** Comparison of students’ perceptions of commercial artificial teeth and 3D-printed teeth.

	Commercial Artificial Teeth	3D-Printed Teeth			
	Median (IQR)	Median (IQR)	Z	*p*	Higher Rated
External anatomy	4 (3–4)	2 (2–3)	−4.241	**<0.001**	**AT**
Internal anatomy	3 (2–3)	3 (3–4)	3.393	**<0.001**	**3DPT**
Location of the pulp chamber	3 (2–4)	3 (2–4)	−0.305	0.760	Comparable
Shape of the pulp chamber	3 (2–4)	3 (2–4)	0.738	0.461	Comparable
Size of the pulp chamber	3 (2–3)	3 (2–3)	0.454	0.650	Comparable
Location of the root canals	3 (2–4)	3 (2–4)	0.046	0.963	Comparable
Shape of the root canals	3 (2–3)	3 (2–4)	1.162	0.245	Comparable
Size of the root canals	3 (2–3)	3 (2–4)	1.468	0.142	Comparable
Radiopacity	3 (2–4)	3 (3–4)	3.422	**<0.001**	**3DPT**
The tactile sensation produced by the turbine and bur during access cavity preparation	2 (1–3)	2 (1–3)	0.090	0.928	Comparable
The sensation of sudden drop upon reaching the pulp chamber	3 (2–4)	2 (1–3)	−1.422	0.155	Comparable
The resistance of the root canal walls during instrumentation	2 (1–3)	3 (2–4)	3.165	**0.002**	**3DPT**
The tactile sensation of the files during instrumentation	2 (2–3)	3 (2–4)	3.316	**<0.001**	**3DPT**
The removal of debris formed during instrumentation, using irrigation.	3 (2–3)	3 (2–4)	0.771	0.441	Comparable
Visibility of the files or verifier on the X-ray	3 (3–5)	4 (3–5)	−0.125	0.901	Comparable

AT—Commercial Artificial Teeth; 3DPT—3D-Printed Teeth; IQR—interquartile range. Values are presented as median (IQR). *p*-Values correspond to comparisons between AT and 3DPT. Comparable indicates no statistically significant differences between groups.

**Table 3:** Students’ perceptions regarding the educational applicability of artificial models (commercial artificial teeth and 3D-printed teeth considered together) in comparison with natural teeth.

Compared to Natural Teeth, Commercial/3D-Printed Artificial Teeth Are More:	Negative		Neutral		Positive		Median (IQR)	*p*
	n	%	n	%	n	%		
Suitable for understanding how to perform non-surgical endodontic treatment.	21	44.7	15	31.9	11	23.4	3 (2–3)	0.309
Suitable for practical endodontics classes.	21	44.7	12	25.5	14	29.8	3 (2–4)	0.630
Suitable for continuous assessment.	18	38.3	7	14.9	22	46.8	3 (2–4)	0.453
Suitable for practical examinations.	17	36.2	10	21.3	20	42.6	3 (2–4)	0.602
More hygienic.	4	8.5	1	2.1	42	89.4	5 (4–5)	**<0.001**

IQR—interquartile range. Values are presented as frequencies (n, %) and median (IQR). Negative responses correspond to scores 1–2, neutral responses to score 3, and positive responses to scores 4–5. *p*-values correspond to comparisons against a neutral distribution.

**Table 4:** Students’ preference ranking for the use of natural teeth, commercial artificial teeth, and 3D-printed teeth in preclinical endodontic training.

	Median (IQR)	Mean Rank	Overall *p*	Post Hoc Comparison	Z	*p*
Natural Teeth	4 (4–5)	2.57	**<0.001**	Natural Teeth vs. Commercial Artificial Teeth	−4.582	**<0.001**
Commercial Artificial Teeth	3 (2–3)	1.51		Natural Teeth vs. 3D-Printed Teeth	−3.410	**<0.001**
3D-Printed Teeth	3 (3–4)	1.91		3D-Printed Teeth vs. Commercial Artificial Teeth	−2.789	**0.005**

IQR—interquartile range. Values are presented as median (IQR) and mean rank. Overall *p*-values correspond to Friedman test comparisons among the three groups. Post hoc pairwise comparisons were performed using the Wilcoxon signed-rank test.

**Table 5:** Evaluators’ perceptions of the similarity of commercial artificial teeth and 3D-printed teeth in comparison to natural teeth.

Similarity to Natural Teeth in Terms of:	Commercial Artificial Teeth		3D-Printed Teeth	
	E1/E2/E3	Median	E1/E2/E3	Median
External anatomy	2/2/4	2	4/4/4	4
Internal anatomy	2/1/2	2	5/5/4	5
Location of the pulp chamber	2/1/4	2	5/5/3	5
Shape of the pulp chamber	2/1/2	2	5/5/5	5
Size of the pulp chamber	2/1/3	2	5/5/5	5
Location of the root canals	3/3/3	3	5/5/4	5
Shape of the root canals	2/2/2	2	5/5/5	5
Size of the root canals	4/3/2	3	5/5/5	5
Radiopacity	2/2/1	2	3/3/1	3
Visibility of the files and verifier on the X-Ray	4/3/5	4	4/3/5	4

E1–E3—evaluators 1 to 3. Values are presented as individual evaluator scores and median values. Higher scores indicate greater perceived similarity to natural teeth.

**Table 6:** Evaluators’ perceptions regarding the educational applicability of artificial models (commercial artificial teeth and 3D-printed teeth considered together) in comparison with natural teeth.

Compared to Natural Teeth, Commercial/3D-Printed Artificial Teeth Are More:	E1/E2/E3	Median
Suitable for understanding how to perform non-surgical endodontic treatment	4/4/2	4
Suitable for practical endodontics classes	4/4/2	4
Suitable for continuous assessment	5/5/5	5
Suitable for practical examinations	5/5/5	5
More hygienic	5/5/5	5

E1–E3—evaluators 1 to 3. Values are presented as individual evaluator scores and median values. Higher scores indicate greater perceived suitability of artificial models compared with natural teeth.

**Table 7:** Evaluators’ preference ranking for the use of natural teeth, commercial artificial teeth, and 3D-printed teeth in preclinical endodontic training.

	E1/E2/E3	Median
Natural Teeth	4/4/5	4
Commercial Artificial Teeth	2/1/3	2
3D-Printed Teeth	4/4/4	4

E1–E3—evaluators 1 to 3. Values are presented as individual evaluator scores and median values. Higher scores indicate greater preference for use in preclinical endodontic training.

**Table 8:** Comparison of evaluators’ scores between commercial artificial teeth and 3D-printed teeth for different endodontic procedures.

	Commercial Artificial Teeth	3D-Printed Teeth	Z	*p*
	Mean ± SD	Mean ± SD		
Access cavity	3.79 ± 1.21	3.97 ± 1.29	−0.955	>0.05
Determination of working length	3.99 ± 1.38	4.25 ± 0.92	−0.702	>0.05
Endodontic preparation	2.84 ± 1.59	3.69 ± 1.20	−3.869	**<0.001**
Verifier fitting	2.96 ± 1.44	3.70 ± 1.12	−4.002	**<0.001**
Root canal filling	2.78 ± 1.46	3.55 ± 0.99	−3.627	**<0.001**

SD—standard deviation. Values are presented as mean ± SD and minimum–maximum range. *p*-Values correspond to comparisons between AT and 3DPT.

**Table 9:** Distribution of procedural errors in commercial artificial teeth and 3D-printed teeth.

	Commercial Artificial Teeth	3D-Printed Teeth
	n (%)	n (%)
Excessive removal of tooth structure during access cavity preparation	20 (42.6%)	10 (21.3%)
Access Perforation	0	0
Over-preparation	0	1 (2.1%)
Under-preparation	31 (66.0%)	13 (27.7%)
Presence of ledge	3 (6.4%)	1 (2.1%)
Presence of debris blockages	28 (59.6%)	8 (17%)
Canal Perforation	1 (2.1%)	0
Apical transportation	1 (2.1%)	1 (2.1%)
Instrument fracture	23 (48.9%)	8 (17.0%)
Overfilling	0	1 (2.1%)
Underfilling	37 (78.7%)	21 (44.7%)
Obturation Voids	22 (46.8%)	6 (12.8%)

**Table 10:** Total number of procedural errors per tooth in commercial artificial teeth and 3D-printed teeth.

	Mean ± SD	Median (IQR)	Z	*p*
Commercially Artificial Teeth	3.53 ± 1.74	4 (2–5)	−8.823	<0.001
3D-Printed Teeth	1.49 ± 1.44	1 (0–2)		

SD—standard deviation; IQR—interquartile range. Values are presented as mean ± SD and median (IQR). *p*-Values correspond to comparisons between AT and 3DPT.

## Data Availability

The data that supports the findings of this study are available from the corresponding author upon reasonable request. The data is not publicly available due to privacy and ethical restrictions (undergoing Ph.D. thesis).

## Supplementary Materials

The following supporting information can be downloaded at: https://www.mdpi.com/article/10.3390/dj14060346/s1, Table S1. Original Spanish version of the students’ questionnaire. Table S2. Test–retest reliability of questionnaire items assessed using weighted Cohen’s kappa coefficients. Table S3. Inter-examiner reliability for the evaluation of endodontic procedures assessed using the intraclass correlation coefficient (ICC). Table S4. Inter-examiner agreement for the identification of procedural errors assessed using Fleiss’ kappa coefficient.

## Abbreviations

The following abbreviations are used in this manuscript:

NT	Natural Tooth
AT	Commercial Artificial Teeth
3D	Three-dimensional
3DPT	Three-dimensional Printed Teeth
