# Supplementary material for: From Research to Education: When Natural Teeth Are the Only Reference—Student Perceptions of PolyJet™ 3D-Printed Teeth in Endodontic Training
Source: Dent J (Basel). 2026 Jun 5;14(6):346. doi: 10.3390/dj14060346 (PMC13297898; doi:10.3390/dj14060346)
Supplement: Supplementary file 1 [file dentistry-14-00346-s001.zip › dentistry-4350995-supplementary.pdf]

Table S1. Original Spanish version of the students' questionnaire.

**Los dientes artificiales comerciales son similares a los dientes naturales en cuanto a:** (Utilice una escala de 1 a 5, donde 1 indica *la menor similitud* y 5 indica *la mayor similitud*, señale su valoración)					
	1	2	3	4	5
Anatomía externa					
Anatomía interna					
Localización de la cámara pulpar					
Forma de la cámara pulpar					
Tamaño de la cámara pulpar					
Localización de los conductos radiculares					
Forma de los conductos radiculares					
Tamaño de los conductos radiculares					
Radiopacidad					
Sensación de perforación con turbina y broca durante la realización de la cavidad de acceso					
Sensación de caída al alcanzar la cámara pulpar					
Resistencia de las paredes de los conductos radiculares durante la instrumentación					
Sensación táctil de las limas durante la instrumentación					
Eliminación de los restos formados durante la instrumentación, con irrigación.					
Visibilidad de las limas o de los verificadores en la radiografía					
**Los dientes impresos en 3D son similares a los dientes naturales en cuanto a:** (Utilice una escala de 1 a 5, donde 1 indica *la menor similitud* y 5 indica *la mayor similitud*, señale su valoración)					
	1	2	3	4	5
Anatomía externa					
Anatomía interna					
Localización de la cámara pulpar					
Forma de la cámara pulpar					
Tamaño de la cámara pulpar					
Localización de los conductos radiculares					
Forma de los conductos radiculares					
Tamaño de los conductos radiculares					
Radiopacidad					
Sensación de perforación con turbina y broca durante la realización de la cavidad de acceso					
Sensación de caída al alcanzar la cámara pulpar					
Resistencia de las paredes de los conductos radiculares durante la instrumentación					
Sensación táctil de las limas durante la instrumentación					
Eliminación de los restos formados durante la instrumentación, con irrigación.					
Visibilidad de las limas o de los verificadores en la radiografía					

**En comparación con los dientes naturales, los dientes artificiales comerciales / impresos en 3D son más**: (Utilice una escala de 1 a 5, donde 1 indica *menos adecuados* y 5 indica *más adecuados*, señale su valoración)					
	1	2	3	4	5
Adecuados para comprender cómo realizar un tratamiento endodóntico no quirúrgico.					
Adecuados para las clases prácticas de endodoncia.					
Justos para el proceso de evaluación continua					
Adecuados para exámenes prácticos					
Más Higiénicos					
**Por favor, clasifique los dientes según su preferencia para su uso en las clases preclínicas de endodoncia** (Utilice una escala de 1 a 5, donde 1 indica menor preferencia para su uso en las clases preclínicas de endodoncia, mientras que 5 indica mayor preferencia.)					
	1	2	3	4	5
Dientes Naturales					
Dientes Artificiales Comerciales					
Dientes Impresos en 3D					

Table S2. Test–retest reliability of questionnaire items assessed using weighted Cohen's kappa coefficients.

Question	T0 (Mean±SD)	T1 (Mean±SD)	Weighted Kappa (95%CI)
**Commercial artificial teeth are similar to natural teeth in terms of:**			
External anatomy	3.55± 0.95	3.50±1.00	0.861 (0.706-1.000)
Internal anatomy	2.55±1.15	2.50±1.00	0.841 (0.697-0.985)
Location of the pulp chamber	3.05±0.95	3.15±0.88	0.811 (0.649-0.972)
Shape of the pulp chamber	2.85±0.99	2.65±0.93	0.777 (0.597-0.957)
Size of the pulp chamber	2.95±0.89	3.05±0.95	0.751 (0.563-0.938)
Location of the root canals	3.20±1.06	3.25±0.85	0.686 (0.541-0.831)
Shape of the root canals	2.80±1.11	3.25±0.79	0.718 (0.560-0.876)
Size of the root canals	2.90±1.21	3.05±1.05	0.776 (0.637-0.916)
Radiopacity	3.10±1.21	3.30±1.08	0.882 (0.789-0.975)
The tactile sensation produced by the turbine and bur during access cavity preparation	2.30±1.34	2.50±1.28	0.758 (0.485-1.000)
The sensation of sudden drop upon reaching the pulp chamber	2.90±1.48	3.05±1.40	0.937 (0.880-0.994)
The resistance of the root canal walls during instrumentation	2.15±0.93	2.15±0.93	0.758 (0.514-1.000)
The tactile sensation of the files during instrumentation	2.15±0.93	2.40±0.82	0.706 (0.473-0.939)
The removal of debris formed during instrumentation, using irrigation.	2.70±1.22	2.75±0.91	0.705 (0.533-0.876)
Visibility of the files or verifier on the X-ray	3.50±1.36	3.75±1.02	0.804 (0.693-0.914)
**3D Printed teeth are similar to natural teeth in terms of:**			
External anatomy	2.75±0.97	2.80±0.95	0.857 (0.703-1.000)
Internal anatomy	3.30±1.03	3.30±1.13	0.820 (0.678-0.962
Location of the pulp chamber	3.15±0.93	3.10±1.02	0.753 (0.586-0.919)
Shape of the pulp chamber	3.05±1.00	3.20±1.01	0.870 (0.760-0.981)
Size of the pulp chamber	2.85±0.93	2.80±0.89	0.780 (0.644-0.916)
Location of the root canals	3.35±0.93	3.45±0.89	0.685 (0.399-0.970)
Shape of the root canals	3.35±0.93	3.30±0.81	0.688 (0.525-0.850)
Size of the root canals	3.30±1.13	3.05±1.05	0.806 (0.671-0.941)
Radiopacity	3.55±0.95	3.65±0.93	0.703 (0.510-0.897)
The tactile sensation produced by the turbine and bur during access cavity preparation	2.55±1.19	2.85±1.23	0.895 (0.796-0.994)
The sensation of sudden drop upon reaching the pulp chamber	2.55±1.23	2.75±1.16	0.784 (0.588-0.979)
The resistance of the root canal walls during instrumentation	3.00±0.97	3.15±1.09	0.878 (0.761-0.995)
The tactile sensation of the files during instrumentation	3.10±1.02	3.20±1.06	0.757 (0.575-0.939)
The removal of debris formed during instrumentation, using irrigation.	2.85±1.14	3.00±1.03	0.889 (0.800-0.978)
Visibility of the files or verifier on the X-ray	3.50±1.32	3.70±1.03	0.815 (0.679-0.951)
**Compared to natural teeth, commercial/3D printed artificial teeth are more**			
Suitable for understanding how to perform non-surgical endodontic treatment.	3.10±1.48	3.30±1.46	0.879 (0.807-0.952)
Suitable for practical endodontics classes.	3.05±1.23	3.25±1.25	0.866 (0.766-0.965)
Suitable for continuous assessment	3.25±1.52	3.45±1.32	0.845 (0.726-0.964)
Suitable for practical examinations	3.10±1.55	3.25±1.48	0.920 (0.865-0.976)
More hygienic	4.50±0.95	4.50±1.00	0.833 (0.606-1.000)
**Please rank the teeth according to your preference for use in pre-clinical endodontics classes**			
Natural teeth	4.35±0.81	4.25±0.97	0.934 (0.863-1.000)
Commercial artificial teeth	3.05±1.05	3.20±1.15	0.807 (0.687-0.927)
3D-printed teeth	3.70±0.92	3.65±0.93	0.909 (0.803-1.000)

[1] T0—initial questionnaire response; T1—questionnaire response after one week; CI—confidence interval.

Values are presented as mean ± standard deviation (SD) and weighted Cohen's kappa (95% CI).

Table S3. Inter-examiner reliability for the evaluation of endodontic procedures assessed using the intraclass correlation coefficient (ICC).

Evaluated procedure	ICC (95% CI)	AT ICC (95% CI)	3DPT ICC (95% CI)
Access cavity	0.962 (0.947-0.974)	0.971 (0.954-0.983)	0.954 (0.926-0.973)
Determination of working length	0.965 (0.951-0.975)	0.975 (0.959-0.985)	0.943 (0.908-0.966)
Endodontic preparation	0.976 (0.966-0.983)	0.986 (0.978-0.992)	0.953 (0.920-0.973)
Verifier fitting	0.965 (0.950-0.976)	0.976 (0.962-0.986)	0.940 (0.898-0.966)
Root canal filling	0.947 (0.925-0.964)	0.977 (0.961-0.987)	0.873 (0.805-0.923)

[1] ICC—intraclass correlation coefficient; CI—confidence interval; AT—commercially artificial teeth; 3DPT—3D-printed teeth. ICC values are presented with 95% confidence intervals.

Table S4. Inter-examiner agreement for the identification of procedural errors assessed using Fleiss' kappa coefficient.

Endodontic Error	Fleiss' κ (95% CI)	p
Excessive removal of tooth structure during access cavity preparation	0.889 (0.772-1.006)	<0.001
Access Perforation	NA	
Over-preparation	1.000 (0.883-1.117)	<0.001
Under-preparation	1.000 (0.883-1.117)	<0.001
Presence of ledge	1.000 (0.883-1.117)	<0.001
Presence of debris blockages	1.000 (0.883-1.117)	<0.001
Canal Perforation	1.000 (0.883-1.117)	<0.001
Apical transportation	1.000 (0.883-1.117)	<0.001
Instrument fracture	1.000 (0.883-1.117)	<0.001
Overfilling	1.000 (0.883-1.117)	<0.001
Underfilling	0.985 (0.868-1.102)	<0.001
Obturation Voids	0.949 (0.832-1,065)	<0.001

[1] κ—Fleiss' kappa coefficient; CI—confidence interval. Values are presented as Fleiss' κ (95% CI). NA—not applicable due to the absence of observed events for the corresponding error category.
